# The Gut-Eye Axis and Microbiome in Ophthalmic Diseases: A Narrative Review

**DOI:** 10.3390/jcm15103563

**Published:** 2026-05-07

**Authors:** Kinga Szymańska, Karolina Sałasińska, Agnieszka Młynarczyk, Justyna Miszczak, Weronika Dmoch, Piotr Maciejewicz

**Affiliations:** 1 Ophthalmology Student Research Group, Department of Ophthalmology, Medical University of Warsaw, 4 Lindleya Street, 02-005 Warsaw, Poland; 2 Department and Clinic of Ophthalmology, University Clinical Center, Medical University of Warsaw, 4 Lindleya Street, 02-005 Warsaw, Poland

**Keywords:** gut-eye axis, microbiome, dysbiosis, short-chain fatty acids, blood-retinal barrier, uveitis, age-related macular degeneration, diabetic retinopathy, glaucoma, dry eye disease, Sjögren’s syndrome

## Abstract

The gut microbiome regulates host metabolism, barrier integrity, and immune homeostasis through microbe–host signaling and bioactive metabolites. Growing evidence suggests that dysbiosis may also influence ocular immune privilege and blood–retinal barrier stability, supporting the emerging concept of the gut–eye axis. This narrative review aimed to integrate retinal, uveal, and ocular surface disorders within a shared functional framework, with emphasis on recurring mechanistic pathways and their translational relevance rather than on single diseases or isolated taxonomic findings. The review was based on a literature search of PubMed and Scopus and primarily included English-language studies published between 2015 and 2025, with earlier seminal papers included when needed. The search was last updated in March 2026, and 101 sources were included in the final narrative synthesis. Across age-related macular degeneration, diabetic retinopathy, glaucoma, uveitis, dry eye disease, and Sjögren’s syndrome, the most consistent microbiome-related signals were functional rather than taxonomic. Recurrent mechanistic themes included Th17/Treg immune programming, barrier dysfunction with microbial product translocation, and systemic metabolite signaling, particularly involving short-chain fatty acids, bile acid receptor pathways, and tryptophan-derived metabolites. Age-related macular degeneration and diabetic retinopathy showed the strongest multi-layered support, whereas uveitis provided a compelling immune-centered biological model that remains limited by treatment-related confounding in human studies. In glaucoma and ocular surface disease, evidence supports biological plausibility, especially in relation to neuroinflammation, mucosal immune dysregulation, and metabolite-dependent anti-inflammatory pathways, although much of the available human literature remains associative. Overall, current evidence supports dysbiosis as a disease modifier that may influence ocular inflammation, angiogenesis, neurodegeneration, and barrier stability. However, clinical translation remains limited by cohort heterogeneity, methodological variability, and incomplete control of confounding factors. Further progress will depend on longitudinal multi-omics cohorts and controlled intervention trials focused on actionable microbial functions.

## 1. Introduction

The gut microbiota is the community of microorganisms that inhabits the intestine, whereas the gut microbiome refers to their collective genetic content and functional activity [1,2,3]. Both are now recognized as important regulators of human health. Their role extends well beyond digestion. They influence metabolism, help maintain epithelial barriers, and shape immune responses [1,2,3]. When this balance is disturbed, dysbiosis develops, and this state has been linked to a broad range of chronic metabolic, autoimmune, and neuroinflammatory disorders [2,3]. Interest in communication between the gut and distant organs has grown rapidly in recent years. The Gut–Brain axis and the gut-skin axis are already well established. More recently, similar attention has been given to the eye, which has led to the concept of the gut-eye axis [4,5,6]. This idea is biologically plausible because changes in the intestinal microbiota can alter systemic immune tone, weaken barrier integrity, and modify the production of bioactive metabolites [1,2,3]. These include short-chain fatty acids and other microbiota-derived signaling molecules [7,8]. The eye may be especially sensitive to such systemic signals. It has high metabolic demands, rich vascularization, and tightly regulated immune control. Under normal conditions, these features protect ocular tissues from excessive inflammation. During systemic immune imbalance, however, this protection may become less effective. In that setting, gut-derived inflammatory and metabolic signals may contribute to retinal, uveal, and ocular surface disease [4,5,6,9,10]. This is particularly relevant in ophthalmology because many major eye diseases are not purely local disorders. They develop through an interplay of systemic inflammation, vascular dysfunction, metabolic stress, and tissue-specific immune responses [4,5,6]. For this reason, the gut-eye axis should not be understood as a simple one-way pathway between the intestine and the eye. Rather, it is a useful framework for explaining how gut-derived immune and metabolic signals may shape ocular vulnerability in patients who already carry genetic, inflammatory, metabolic, or age-related risk factors [4,5,6,9,10]. Another important point is that microbiome-related disease associations are often clearer at the functional level than at the taxonomic level. The same genus may appear relevant in one cohort and not in another, depending on diet, age, medication use, geography, and sequencing methods. By contrast, changes in short-chain fatty acid production, barrier regulation, bile acid signaling, or inflammatory tone may be easier to compare across studies and easier to interpret biologically [7,8,11,12,13].

This is especially important in ophthalmology, where cohorts are often small, and disease phenotypes are heterogeneous. Unlike previous reviews, which often focus on single ophthalmic diseases or primarily descriptive taxonomic findings, the present review examines whether common functional mechanisms recur across retinal, uveal, and ocular surface disorders. In particular, we focus on microbial metabolites, barrier dysfunction, endotoxin-related inflammatory signaling, and immune programming as cross-cutting pathways that may link gut dysbiosis to ocular pathology. By emphasizing cross-disease mechanistic integration and translational interpretation rather than disease-by-disease taxonomic description, this review aims to clarify what is currently biologically plausible, what remains uncertain, and which signals may be most relevant for future clinical translation.

## 2. Materials and Methods

This narrative review was designed to provide a functional overview of the gut–eye axis across retinal, uveal, and ocular surface disorders. The aim was not to address a single narrowly defined clinical question, but to integrate evidence from multiple ophthalmic conditions within a shared mechanistic framework. A narrative approach was selected because the field remains broad and methodologically heterogeneous, with relevant evidence derived from observational studies, experimental models, genetic analyses, and translational research.

The literature search was conducted in PubMed and Scopus. We primarily included English-language studies published between 1 January 2015 and 31 December 2025. The search was last updated in March 2026. Earlier seminal studies were additionally included when needed to support key background concepts, including ocular immune privilege, blood–retinal barrier biology, intestinal permeability, and microbiome–host signaling. In total, 101 sources were included in the final narrative synthesis.

**Figure 1:**
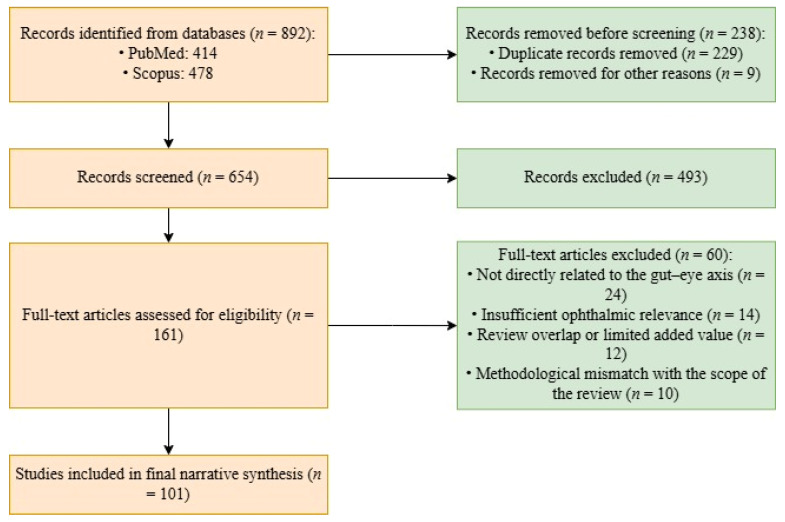
A flow diagram of the study selection process.

The search strategy combined terms related to the gut microbiome, ophthalmic diseases, and relevant mechanistic pathways. The main search phrases included “gut–eye axis”, “gut microbiome and ocular diseases”, “intestinal dysbiosis and eye diseases”, “retina and gut microbiota”, “uveitis and gut microbiome”, “glaucoma and gut microbiota”, “dry eye disease and gut microbiota”, “age-related macular degeneration and gut microbiome”, “diabetic retinopathy and gut microbiota”, “microbial metabolites and retina”, and “gut permeability and ocular inflammation”. Boolean operators were used to refine the search. The study selection process is shown in Figure 1.

A total of 892 records were identified through database searching (PubMed: 414; Scopus: 478). Before screening, 238 records were removed, including 229 duplicate records and 9 records removed for other reasons. The remaining 654 records underwent title and abstract screening, and 493 were excluded at this stage. Full texts of 161 articles were then assessed for eligibility. After full-text evaluation, 60 articles were excluded for the following reasons: not directly related to the gut–eye axis (*n* = 24), insufficient ophthalmic relevance (*n* = 14), review overlap or limited added value (*n* = 12), and methodological mismatch with the scope of the review (*n* = 10). Ultimately, 101 sources were retained for the final narrative synthesis.

For the purposes of this review, primary studies were defined as original reports presenting new data, including observational human studies, experimental animal studies, genetic analyses, interventional studies, and selected case-based reports when directly relevant to the topic. Review articles and meta-analyses were used mainly for broader context and mechanistic interpretation rather than as the principal evidentiary basis. Conference abstracts were not included. Very small case series were not treated as a major basis for interpretation unless they addressed a specific issue not covered elsewhere in the literature.

During interpretation, particular attention was paid to heterogeneity across studies. Differences between reports may reflect variation in diet, age, geography, medication exposure, comorbidities, disease phenotype, sequencing methodology, and bioinformatic pipelines. In ocular surface studies, the specific challenges of low-biomass sampling, including contamination risk and batch effects, were also taken into account.

As a narrative review, this work has inherent limitations. We did not perform a formal quantitative synthesis or apply a structured risk-of-bias tool. However, greater interpretive weight was given to findings supported by more than one line of evidence, especially when observational findings were reinforced by experimental or genetic data. This approach was used to emphasize signals that appear more biologically coherent across the available literature.

## 3. Functional Basis of the Gut-Eye Axis

**Figure 2:**
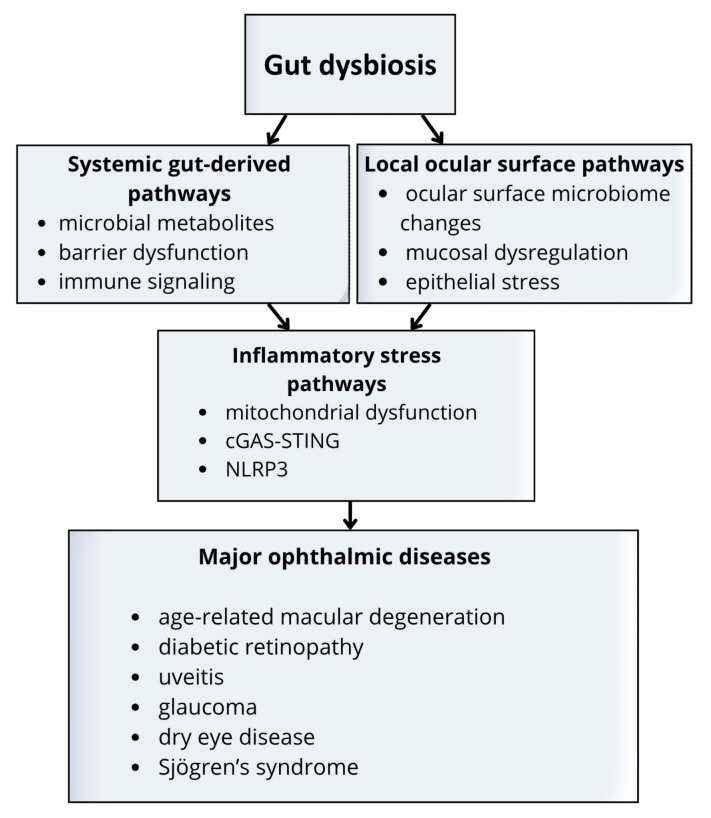
Conceptual framework of the gut-eye axis across major ophthalmic diseases. Gut dysbiosis may influence ocular pathology through two partially overlapping routes. Systemic gut-derived pathways include microbial metabolites, barrier dysfunction, and immune signaling, which are most relevant to retinal and uveal disease. Local pathways involve ocular surface microbiome changes, mucosal dysregulation, and epithelial stress, which are particularly relevant to dry eye disease and Sjögren’s syndrome. These mechanisms may converge on downstream inflammatory stress pathways, including mitochondrial dysfunction, cGAS-STING signaling, and NLRP3 inflammasome activation, which may further amplify tissue vulnerability. Together, these pathways may contribute to age-related macular degeneration, diabetic retinopathy, uveitis, glaucoma, dry eye disease, and Sjögren’s syndrome.

The gut-eye axis can be understood through two distinct but overlapping routes: systemic and local. Systemic pathways involve gut-derived signals, such as metabolites, inflammatory mediators, and immune programming, that influence ocular tissues through the circulation. These mechanisms are most relevant to retinal and uveal disease. Local pathways involve the ocular surface microbiome and its interaction with mucosal immunity, epithelial integrity, and tear film homeostasis. This distinction is especially relevant in the eye, where tissue homeostasis depends on tightly regulated barrier and immune control [9,10]. The main systemic and local components of the gut-eye axis are summarized schematically in Figure 2.

Across this field, microbial function often appears more informative than taxonomic composition alone. Similar functional patterns may be seen across different cohorts, even when microbial signatures are not identical. This is especially important in ophthalmology, where studies are often small and heterogeneous. Taxonomic associations are frequently context-dependent and may vary with diet, host metabolism, medication exposure, geography, and sequencing methods. By contrast, functional pathways such as reduced short-chain fatty acid production, altered bile acid signaling, and barrier dysfunction are often easier to interpret biologically and more suitable for cross-disease comparison [7,8,11,12,13].

### 3.1. Microbial Metabolites and Immune Regulation (Systemic)

Short-chain fatty acids are among the most important products of the gut microbiome. Acetate, propionate, and butyrate are generated during fiber fermentation and help maintain intestinal epithelial integrity while also regulating immune responses [7,8]. Their effects are generally anti-inflammatory, as they support regulatory T-cell activity and help limit excessive inflammatory responses [8]. Butyrate is also an important energy source for colonocytes, which directly links microbial fermentation to epithelial health. From a mechanistic perspective, short-chain fatty acids act through several complementary routes. They can signal through free fatty acid receptors such as FFAR2 and FFAR3, and they can also influence gene expression through histone deacetylase inhibition [7,8]. Through these pathways, they affect epithelial integrity, cytokine production, immune cell differentiation, and inflammatory tone. For this reason, short-chain fatty acids are increasingly viewed not only as metabolic by-products but also as active mediators connecting diet, microbial ecology, and host physiology.

Evidence that these effects extend to the eye comes mainly from experimental studies. In endotoxin-induced uveitis, short-chain fatty acids were detected in ocular tissue and reduced inflammatory responses. In autoimmune uveitis models, oral short-chain fatty acids, especially propionate, reduced disease severity and were associated with stronger regulatory responses in ocular-draining lymphoid tissues [14,15]. These findings support the idea that gut-derived metabolites can influence ocular immunity without direct microbial invasion of ocular tissue. Immune programming is one of the clearest examples of this principle. Gut microbes influence the balance between effector and regulatory responses, including the relationship between Th17 cells and regulatory T cells. This balance is highly relevant to immune-mediated eye disease because excessive Th17 activity can promote chronic inflammation, whereas regulatory pathways help maintain tolerance. Short-chain fatty acids, especially butyrate and propionate, are often linked to stronger regulatory responses, whereas dysbiosis may favor a more inflammatory set point [8,14,15]. In this way, the gut microbiome may affect not only whether inflammation occurs, but also how easily it becomes amplified or sustained once triggered.

Other metabolite pathways may also contribute to the gut-eye axis. Gut microbes modify bile acid composition and thereby alter host receptor signaling. Primary bile acids are converted into secondary bile acids by microbial enzymes, which changes the composition of the circulating bile acid pool. This matters because bile acids act as signaling molecules, not just digestive components. In experimental diabetic retinopathy, activation of the bile acid receptor TGR5 reduced vascular leakage and inflammatory activation [13]. This provides a plausible link between microbial metabolism and retinal vascular stability. Tryptophan-derived microbial metabolites may provide another link between the gut and the eye. The tryptophan pathway lies at the intersection of metabolism, immunity, and neurobiology. Microbial products derived from tryptophan can influence aryl hydrocarbon receptor signaling and modulate inflammatory responses. Although evidence remains strongest in experimental systems, this pathway may help explain how the microbiome influences both immune regulation and neurodegeneration.

Overall, the metabolite-centered view of the gut-eye axis is valuable for two reasons. First, it offers a more stable framework than taxonomic associations alone. Second, it has clearer translational potential because metabolites and their receptors may be easier to measure and target than whole microbial communities. At the same time, caution is needed. A given circulating metabolite may reflect diet, host metabolism, microbial activity, or all three. For this reason, future studies will need integrated microbiome and metabolomic data rather than indirect inference from taxonomy alone. Despite strong experimental data, several questions remain unresolved. It is not yet clear to what extent gut-derived short-chain fatty acids reach human ocular tissues in biologically meaningful concentrations. Most direct evidence comes from animal models, where metabolite levels are easier to measure, and experimental conditions can be tightly controlled. In humans, the relationship between fecal or circulating short-chain fatty acid levels and ocular health remains largely indirect. Another important issue is target specificity. Receptors such as FFAR2 and TGR5 are plausible downstream mediators, but their functional relevance in human ocular tissues has not yet been established with sufficient clarity. It is also possible that the effects of microbial metabolites are context-dependent. A pathway that is protective under conditions of low-grade inflammation may behave differently in an acute or highly cytokine-driven environment. Future work will need to define these molecular targets more precisely and clarify which metabolite-mediated signals are truly relevant in human disease.

### 3.2. Barrier Dysfunction and Systemic Inflammatory Signaling

A second major mechanism involves intestinal barrier dysfunction. Dysbiosis may weaken epithelial integrity, increase intestinal permeability, and promote chronic low-grade inflammation [11,12]. This process is often discussed in relation to endotoxemia, in which microbial products that are normally contained within the gut lumen gain greater access to the circulation. Even modest increases in exposure to these signals may shape systemic inflammatory tone over time. Lipopolysaccharide is particularly important in this process because it activates innate immune responses through TLR4 and may affect distant tissues, including the retina [11,12,16]. In experimental settings, lipopolysaccharide has been shown to activate inflammatory pathways in the retina and to trigger endothelial TLR4-dependent microglial activation [16]. This provides a plausible route by which gut-derived inflammatory signals could influence retinal homeostasis.

Barrier-related signaling may also affect the blood-retinal barrier more directly. Experimental studies have shown that lipopolysaccharide exposure can impair protective transport at the inner blood-retinal barrier, including reduced P-glycoprotein function [17]. This is relevant because retinal health depends not only on limiting inflammatory cell entry, but also on maintaining tightly regulated exchange across the barrier. Once this system is weakened, retinal tissue may become more vulnerable to circulating inflammatory signals and metabolic stress. The same logic applies to barrier biology more broadly. Intestinal permeability is not a simple on-or-off phenomenon. It reflects continuous interaction between epithelial cells, the mucus layer, microbial metabolites, diet, and host immune signaling. Once this balance is disturbed, greater exposure to microbial products may contribute to chronic systemic inflammation [11,12]. In susceptible ocular tissues, such persistent signaling may increase the likelihood of barrier dysfunction, microglial activation, endothelial stress, and impaired tissue repair [16,17]. This helps explain why dysbiosis may act as a modifier across several apparently distinct ophthalmic diseases.

Gut microbes also shape bile acid composition by converting primary into secondary bile acids, which changes host receptor signaling. In diabetic retinopathy models, activation of the bile acid receptor TGR5 improved vascular stability and reduced inflammatory changes [13]. This provides another example of how gut-derived signals may influence the retinal environment through pathways that combine metabolic and inflammatory regulation.

Overall, this framework supports the idea that intestinal barrier disruption may translate into ocular vulnerability through sustained inflammatory signaling and reduced barrier resilience. It also helps explain why dysbiosis often behaves less like a single disease trigger and more like an amplifier of pre-existing susceptibility. The interaction between gut and ocular barriers may follow a “double-hit” model. In this framework, intestinal permeability first promotes systemic exposure to microbial products, and these circulating signals then weaken blood-retinal barrier resilience, making ocular tissues more vulnerable to inflammatory and metabolic stress. This is a useful conceptual model, but it remains incompletely defined. In particular, the threshold of lipopolysaccharide exposure required to affect human ocular tissues is not known, and it is still unclear whether lipopolysaccharide acts as a primary trigger or mainly amplifies pre-existing inflammation. Current experimental models also have important limitations, as they often rely on acute or high-dose lipopolysaccharide exposure that may not reflect the chronic, low-grade inflammatory state associated with human dysbiosis. Identifying the specific downstream targets of endotoxin-related signaling at the blood-retinal barrier remains an important priority for future research.

### 3.3. Local Ocular Surface Microbiome and Mucosal Immune Dysregulation

Local ocular surface mechanisms should be distinguished from systemic gut-derived pathways. At the ocular surface, the main issues concern low-biomass microbial ecology, mucosal immune regulation, epithelial stress, and tear film instability. These pathways are most relevant to dry eye disease and Sjögren’s syndrome rather than to retinal disease.

Human sequencing studies support the presence of a low-biomass ocular surface microbiome [18]. At the same time, the interpretation of these data remains challenging. In low-biomass environments, even small differences in sampling technique, contamination, or data processing may influence apparent community structure. For this reason, the most reliable conclusions usually concern broader shifts in diversity or ecological instability rather than the presence of a single disease-defining taxon. This point is especially relevant in dry eye disease and autoimmune ocular surface disorders. In these settings, the key signal is often ecological instability rather than a single microorganism. Local changes in microbial diversity may interact with impaired mucosal immunity, epithelial stress, and altered tear film composition. These local processes may also reflect broader systemic immune changes, particularly in diseases such as Sjögren’s syndrome.

The origin and stability of the ocular surface microbiome remain unresolved. It is still unclear whether the microorganisms detected at the ocular surface represent stable residents, fluctuating communities, or mainly transient colonizers derived from the environment. This distinction is important because it affects how local dysbiosis should be interpreted and how realistic microbiome-directed therapies may be. If these microbial communities are highly transient, local interventions may have only limited or short-lived effects. In addition, the connection between the gut and the ocular surface is likely mediated, at least in part, through the common mucosal immune system, but the relevant trafficking pathways between the gut and the lacrimal functional unit are not yet well defined in humans. Most mechanistic models in this area still depend heavily on animal data, and their relevance to human ocular surface disease remains to be clarified. Addressing these uncertainties will be important for developing more targeted strategies in dry eye disease and Sjögren’s syndrome.

### 3.4. Downstream Ocular Inflammatory Stress Pathways

Beyond local ocular surface dysbiosis, systemic microbial imbalance may also contribute to broader downstream inflammatory stress pathways in ocular tissues. Proposed mechanisms include mitochondrial dysfunction, cGAS-STING signaling, and NLRP3 inflammasome activation [19,20,21,22]. These pathways are not specific to the ocular surface. Rather, they represent more general stress responses that may affect retinal or other ocular tissues after systemic immune or metabolic disturbance. Mitochondria are central to cellular energy metabolism, but they also participate in innate immune regulation. When mitochondrial homeostasis is disturbed, inflammatory signaling may become amplified. This is particularly relevant in tissues with high metabolic demand, such as the retina. The cGAS-STING pathway provides another possible bridge. It senses cytosolic DNA and drives type I interferon signaling. In principle, this pathway may connect cellular stress, inflammation, and vascular dysfunction, all of which are relevant to retinal disease. Likewise, inflammasome activation may contribute to chronic inflammatory amplification in settings of persistent stress. Although this area remains less established than the short-chain fatty acid and lipopolysaccharide pathways, it offers a useful framework for understanding how chronic inflammatory stress may contribute to retinal damage and neurodegeneration. At present, however, evidence linking these pathways directly to the gut-eye axis remains predominantly experimental and mechanistic rather than firmly established in human ophthalmic cohorts.

Taken together, these observations support a layered model of the gut-eye axis. Systemic pathways appear most relevant to retinal and uveal disease, whereas local microbiome-associated pathways are most relevant to ocular surface disorders. Downstream inflammatory stress responses may further amplify tissue vulnerability across both contexts.

## 4. Disease-Specific Evidence

The relevance of gut dysbiosis does not appear to be uniform across ophthalmic diseases. In some conditions, support comes from several complementary lines of evidence, including human cohort studies, genetic analyses, metabolomic data, and experimental models. In others, the literature remains mainly associative or depends heavily on animal work. Interpretation is further complicated by differences in age, diet, disease stage, medication exposure, geography, and microbiome methodology. These issues should be kept in mind throughout the following sections.

### 4.1. Age-Related Macular Degeneration

Age-related macular degeneration is one of the ophthalmic diseases with the strongest support for the gut-eye axis involvement. Human studies have reported differences in gut microbiota composition in patients with neovascular age-related macular degeneration, and metagenomic analyses suggest a relatively consistent disease-associated microbial pattern across independent cohorts [23,24]. These findings do not point to a single disease-specific organism. Rather, they suggest a broader microbial configuration associated with disease status. However, causal inference from observational human data remains limited. A more recent pilot study that combined fecal microbiome profiling with short-chain fatty acid and bile acid metabolomics also identified stage-related differences across AMD groups, which further supports a function-oriented interpretation of gut-eye interactions in this disease [25].

The human data are further supported by genetic epidemiology. Mendelian randomization studies suggest that selected gut microbial features may be linked to susceptibility to age-related macular degeneration, and mediation analyses raise the possibility that microbiome-derived metabolites contribute to part of this association [26,27,28,29]. Although Mendelian randomization provides a more causally informative framework than conventional observational studies, its interpretation still depends on important assumptions, including the possibility of horizontal pleiotropy. Additional metabolite-focused causal analyses have also suggested that circulating metabolic intermediates may contribute to AMD susceptibility [30].

Experimental findings broadly support the same direction. In germ-free mice, the absence of gut microbiota was associated with transcriptomic changes in the retinal pigment epithelium and choroid relevant to age-related macular degeneration pathobiology, together with reduced choroidal neovascularization [31]. Recent reviews have therefore identified age-related macular degeneration as one of the most promising ophthalmic conditions for microbiome-oriented research [32]. A recent bioinformatic study also linked gut dysbiosis to AMD progression, although this approach remains hypothesis-generating [33].

This overall picture is biologically credible. Age-related macular degeneration involves chronic low-grade inflammation, dysfunction of the retinal pigment epithelium-Bruch membrane-choroid complex, and, in neovascular disease, pathologic angiogenesis. These processes may all be influenced by systemic inflammatory tone and metabolite signaling. That does not mean the gut microbiome is the primary cause of the disease. A more cautious interpretation is that dysbiosis may modify susceptibility, progression, or the balance between protective and damaging inflammatory responses.

Mitochondrial stress and innate immune activation may also be relevant, particularly in the aging retina. This matters because aging itself is closely linked to both microbiome change and retinal vulnerability. In that sense, the gut-eye axis in age-related macular degeneration may be better understood as part of a broader aging-associated inflammatory network than as an isolated disease pathway.

Most human studies, however, remain cross-sectional and are still vulnerable to confounding by age, diet, cardiometabolic disease, and medication exposure. Even with these limitations, age-related macular degeneration remains one of the strongest candidates for future microbiome-based stratification and intervention studies in ophthalmology because the observational, genetic, and experimental data are relatively coherent.

### 4.2. Diabetic Retinopathy

In diabetic retinopathy, dysbiosis is best viewed as a modifier of metabolic and vascular injury rather than an isolated cause of disease. Clinical and translational studies suggest that patients with diabetic retinopathy show altered gut microbiota composition, often with reduced abundance of short-chain fatty acid-producing bacteria and enrichment of potentially pro-inflammatory taxa [34,35,36,37,38,39]. This pattern fits a broader model of increased intestinal permeability, chronic inflammatory activation, and vascular stress. A recent systematic review and meta-analysis broadly supported this direction, while also highlighting substantial heterogeneity across available cohorts [38]. Several studies support this framework. In diabetic mice, intermittent fasting altered gut microbiome structure and reduced retinopathy severity [40]. In patients with type 2 diabetes, probiotic treatment reduced bacterial translocation, suggesting that barrier dysfunction may be modifiable in humans [41]. Experimental work has also linked retinal injury in diabetes to bile acid signaling, with activation of the bile acid receptor TGR5 reducing vascular leakage and inflammatory activation in diabetic retinopathy models [13].

These observations are mechanistically appealing because they align with established features of diabetic retinopathy. The disease is closely linked to chronic metabolic stress, endothelial dysfunction, inflammation, and barrier breakdown. From this perspective, the gut-eye axis adds a systemic layer to processes that are already known to be central to disease biology. It suggests that gut-derived signals may amplify established pathogenic mechanisms rather than create a separate pathway altogether. One recurring theme is loss of protective microbial function. Reduced abundance of short-chain fatty acid-producing organisms may remove an anti-inflammatory influence, while increased permeability may allow more inflammatory signaling to reach the circulation. Together, these changes would be expected to worsen retinal endothelial stress and blood-retinal barrier dysfunction. In this sense, dysbiosis may help connect metabolic disease to vascular inflammation at a systems level.

Genetic analyses point in a similar direction. Mendelian randomization studies have explored links between inflammatory bowel disease and diabetic retinopathy, supporting the idea of broader gut-retina crosstalk [42]. Still, most human studies evaluate only one level of the pathway at a time. Barrier markers, microbiome features, systemic metabolites, and retinal outcomes are rarely measured together, which limits interpretation. More recent longitudinal data also suggest that gut microbiome features may have predictive value for retinopathy risk in patients with diabetes, although these findings still require validation across independent cohorts [43]. Metabolomic and lipidomic studies further suggest that systemic metabolic signatures may help stratify diabetic retinopathy stage and risk, although these findings still require external validation [44,45,46].

Treatment-related confounding is another major issue. Antidiabetic therapies such as metformin, SGLT2 inhibitors, and GLP-1 receptor agonists are known to influence gut microbial composition and host metabolism. In many currently available cohorts, medication exposure is not described in enough detail to determine whether observed microbiome differences reflect diabetic retinopathy itself, diabetes severity, treatment effects, or a combination of these factors.

Even with these limitations, diabetic retinopathy remains one of the conditions in which the gut-eye axis appears especially plausible, largely because the proposed mechanisms fit well with the known inflammatory and vascular features of the disease.

### 4.3. Uveitis

Uveitis is best understood as an immune-dominant disease in which the microbiome may shape inflammatory thresholds rather than act as a single direct trigger. Experimental and review-based evidence supports the idea that intestinal immune programming can influence ocular inflammation through changes in effector and regulatory responses [47,48,49,50]. This is particularly relevant to Th17-related pathways, which play a central role in many forms of non-infectious uveitis and are closely linked to IL-23/IL-17 signaling [51,52]. Experimental autoimmune uveitis models provide the strongest evidence for a gut-dependent component. Oral antibiotics, germ-free conditions, dietary intervention, and metabolite-based approaches can all alter disease severity in animals [5,14,15,49]. These findings suggest that gut microbial composition and function may influence how pathogenic immune responses are generated, maintained, or trafficked. Additional experimental work suggests that PD-L1-related immune perturbation may also reshape gut microbiota in autoimmune uveitis [53].

Human data are more difficult to interpret. Clinical studies have reported reduced microbial diversity and shifts in gut community structure in non-infectious uveitis, but the specific taxonomic patterns vary across cohorts [48,49,50]. That inconsistency is not surprising. Uveitis includes a heterogeneous group of disorders, and many studies combine patients with different etiologies, anatomical subtypes, and treatment exposures. This makes taxonomic reproducibility difficult, especially when sample sizes are modest. Host genetics likely adds another layer of complexity. HLA-B27-associated anterior uveitis is one example in which interactions between genetic predisposition and gut ecology appear especially plausible [54]. Genetic analyses from related immune-mediated ocular phenotypes, including Behçet’s disease, also support possible links between gut microbial traits and ocular inflammation, although extrapolation to non-infectious uveitis should remain cautious [55].

A major limitation of the human literature is the difficulty of separating disease-related signals from treatment effects. Many patients with chronic non-infectious uveitis receive corticosteroids, methotrexate, or biologic agents, all of which may alter immune tone and potentially influence microbiome-related readouts. As a result, most currently available human studies cannot fully disentangle the effects of uveitis itself from those of immunosuppressive treatment. This concern is reinforced by experimental data showing that antimetabolite drugs can alter intestinal microbiota composition in autoimmune uveitis models [56].

Overall, the literature is mechanistically persuasive, but translation remains limited. Human studies are still small, frequently confounded by treatment, and rarely integrate microbiome sequencing with immune phenotyping and metabolite profiling. At present, the gut-eye axis in uveitis is best viewed as a strong biological model with substantial experimental support, but not yet as a source of stable clinical biomarkers.

### 4.4. Glaucoma

The glaucoma literature suggests that gut dysbiosis may contribute to neuroinflammation and retinal ganglion cell vulnerability, although the evidence is still less developed than in age-related macular degeneration or diabetic retinopathy. In a large human cohort, patients with glaucoma had lower abundance of several butyrate-producing taxa, including *Butyrivibrio*, *Coprococcus*, and members of *Ruminococcaceae* [57]. These taxa were also associated with clinically relevant phenotypes such as lower intraocular pressure and less optic nerve cupping, which makes the finding notable.

Experimental studies support the same general direction. In a rat model of chronic ocular hypertension, glaucoma was associated with marked changes in gut microbiota composition, including shifts in the Firmicutes/Bacteroidetes ratio and changes in several genera linked to retinal ganglion cell loss [58]. In a murine glaucoma model, commensal microflora were shown to prime CD4+ T-cell responses to bacterial heat-shock proteins and thereby promote progressive neurodegeneration [59]. This has been one of the most influential mechanistic findings in the field because it offers a plausible explanation for continued degeneration beyond intraocular pressure alone.

More recent metabolite-centered work adds another layer to this model. Indoleacetic acid, a gut microbiota-derived tryptophan metabolite, reduced neuroinflammation and neurodegeneration in experimental glaucoma through AHR/RAGE-related signaling [60]. This suggests that the microbiome may influence glaucoma not only through immune priming but also through metabolite-driven modulation of inflammatory stress.

At the same time, the interpretation of human glaucoma studies is complicated by important confounders. Aging, systemic disease, and chronic medication exposure may all influence gut microbial composition. In addition, long-term treatment with topical prostaglandin analogs, beta-blockers, and preservative-containing formulations may affect mucosal biology and could potentially influence microbiome-related signals through chronic ocular surface exposure and nasolacrimal drainage. This possibility remains insufficiently discussed in current glaucoma microbiome studies. A recent review of glaucoma and the human microbiome also emphasized that medication exposure and host-level confounding remain major interpretive challenges in this field [61].

Taken together, the findings support biological plausibility, but the human data remain mainly associative. Glaucoma is also clinically heterogeneous, so the same mechanism may not apply equally across all subtypes. For now, the evidence justifies continued investigation, but it does not establish dysbiosis as a confirmed driver of glaucoma progression [62].

### 4.5. Ocular Surface Disease: Dry Eye Disease and Sjögren’s Syndrome

The ocular surface literature differs from the retinal literature because it involves both gut dysbiosis and local microbial instability in a low-biomass environment. In dry eye disease, sequencing studies have shown that the ocular surface microbiome differs from that of healthy controls [63,64]. Studies comparing autoimmune and non-autoimmune dry eye also suggest that local microbial patterns may reflect broader immune context rather than surface irritation alone [65]. More recent work has also emphasized the need for stronger methodological standardization in ocular surface microbiome research, particularly with regard to sampling, sequencing workflows, and low-biomass interpretation [66]. Broader reviews have likewise emphasized the growing relevance of the gut-eye axis in dry eye disease [67].

Experimental work gives this field more mechanistic depth. In a mouse model of dry eye, oral tributyrin reduced ocular surface inflammation, and this effect depended on the epithelial short-chain fatty acid transporter SLC5A8 [68]. This provides an important proof of concept that a gut-derived metabolite can influence a defined host pathway at the ocular surface. More broadly, these findings support the view that dry eye disease should not be understood only as a local surface disorder, but also as part of a wider mucosal and immune network.

Sjögren’s syndrome fits naturally within this broader framework. Patients with primary Sjögren’s syndrome show clear evidence of gut dysbiosis, including reduced abundance of *Bifidobacterium* and butyrate-producing taxa together with increased abundance of *Prevotella* or Proteobacteria-rich profiles [69,70,71,72]. Some of these changes correlate with dry eye severity and systemic immune markers such as Th17/Treg imbalance, reduced FOXP3 expression, and altered IL-10-related regulation [71,72]. Metabolomic studies add further support by showing disturbances in bile acid and amino acid metabolism together with expansion of *Escherichia-Shigella* [73]. More recent Mendelian randomization data also support a causal relationship between selected gut microbial taxa and Sjögren’s syndrome, although this evidence remains indirect with respect to ocular manifestations [74]. At the ocular surface, reduced alpha diversity and disease-specific changes in tear film microbiota have also been reported in Sjögren’s syndrome and in aqueous-deficient dry eye more broadly [75,76]. Recent studies have further expanded this field by linking ocular and gut microbiome profiles to dry-eye severity across severe inflammatory phenotypes and by emphasizing the influence of physiological and environmental factors on ocular surface microbial readouts [77,78,79].

The strength of the ocular surface field lies in the convergence of local ocular surface findings, broader systemic gut microbiome associations, and experimental work suggesting metabolite-dependent anti-inflammatory effects. In contrast to some retinal diseases, where the eye is anatomically distant from the gut, ocular surface disease fits naturally within a mucosal immunology framework. At the same time, this area remains highly sensitive to methodological limitations, especially low microbial biomass, treatment exposure, and the confounding effects of systemic autoimmunity. Even so, ocular surface disease, particularly in its autoimmune form, remains one of the clearest examples of potential gut-eye interaction in ophthalmology.

Across the conditions reviewed above, several patterns emerge. Age-related macular degeneration and diabetic retinopathy show the strongest convergence of clinical, genetic, and experimental evidence, which makes them leading candidates for microbiome-based research in ophthalmology. Uveitis provides a compelling immune-centered model, although human data remain limited by disease heterogeneity and treatment confounding. Glaucoma and ocular surface disease show growing support for metabolite-related and mucosal immune pathways, but the evidence remains largely associative. A common theme across all conditions is that functional signals, particularly those related to short-chain fatty acids, barrier integrity, and immune programming, appear more consistent than taxonomic signatures. Table 1 summarizes the current strength of evidence across diseases, together with the main proposed mechanisms and the principal limitations affecting interpretation.

### 4.6. Critical Appraisal of Current Evidence

The current literature should be interpreted with caution. Differences between studies are often not driven by disease alone, but also by substantial methodological and clinical heterogeneity. Geography matters because it shapes diet, environmental exposure, and baseline microbiome composition. Age is another major source of variation, especially in ophthalmology, where patients often have multiple comorbidities and more complex treatment histories.

Technical differences make interpretation even harder. Many studies still rely on 16S rRNA sequencing, which is useful for broad microbial profiling but offers limited taxonomic resolution and little direct information about microbial function. Shotgun metagenomics and multi-omics approaches may provide a more informative picture, but they remain less common and are not used consistently across cohorts. Medication exposure is also a major confounder. In diabetic cohorts, metformin may substantially alter gut microbial composition. In glaucoma, long-term topical treatment and preservatives may influence microbiome-related signals. Similar concerns apply to immunosuppressive therapy in inflammatory eye disease.

Most available studies are also cross-sectional. This means they can identify associations, but they cannot reliably determine whether dysbiosis precedes disease, follows it, or reflects treatment and comorbidity burden. Better-controlled longitudinal cohorts, together with more consistent integration of microbiome, metabolomic, and clinical data, will be needed to identify which signals are truly robust and clinically meaningful.

## 5. Therapeutic Modulation and Future Directions

The growing interest in the gut-eye axis has naturally raised the question of whether microbiome-related pathways can be targeted therapeutically. At present, the most realistic view is that such strategies are more likely to serve as adjuncts than as replacements for standard ophthalmic care. The strongest support is still found in ocular surface disease and in experimental autoimmune settings. In retinal and neurodegenerative disease, the translational signal is interesting, but still early. Much of the available evidence remains preclinical, and even human studies are often small, heterogeneous, and difficult to compare directly.

### 5.1. Microbiome-Targeted Interventions

The clearest early clinical signal comes from dry eye disease. A randomized controlled trial showed that four months of oral synbiotic supplementation improved dry eye symptoms and helped stabilize tear film parameters compared with placebo [80]. Although the study was small, it remains an important proof of concept that systemic microbiome modulation can influence ocular surface outcomes in humans.

Preclinical studies point in the same direction. In murine dry eye models, probiotic formulations reduced inflammatory markers, improved tear secretion, and altered gut microbial composition [81,82,83,84,85]. These findings are encouraging, but they should not be interpreted as evidence for a generic probiotic effect. Benefits appear to be strain-specific, context-dependent, and model-dependent. What works in one experimental system may not translate well to another, and certainly not automatically to human disease.

Broader manipulation of the microbiome may also influence ocular aging phenotypes. Fecal microbiota transfer between young and aged mice has been shown to reverse several hallmarks of aging in the gut, eye, and brain, which supports the idea that microbiome-directed interventions may eventually have broader ophthalmic relevance [86]. Even so, in conditions such as uveitis and glaucoma, these strategies remain largely preclinical. The main limitation is not a lack of biological plausibility, but a lack of robust human evidence.

Another difficulty is that microbiome modulation is unlikely to work through one universal mechanism. In some settings, benefit may depend mainly on reduced inflammatory signaling. In others, improved barrier function, restoration of microbial diversity, or altered metabolite production may matter more. This makes the field attractive, but also hard to standardize. Differences in probiotic strains, dosing schedules, treatment duration, baseline diet, and host phenotype all complicate interpretation. For now, microbiome-targeted interventions should be regarded as promising but still investigational. Recent reviews of microbiome-based therapeutics in ocular disease have reached a similar conclusion, emphasizing biological promise alongside limited standardization and sparse human data [87]. Additional preclinical work suggests that butyrate-based modulation may also attenuate ocular surface inflammation in meibomian gland dysfunction models [88].

### 5.2. Dietary Modulation

Diet is currently the most practical way to influence the gut-eye axis in clinical settings. The strongest human evidence comes from age-related macular degeneration. Higher adherence to a Mediterranean dietary pattern has been associated with lower risk of advanced age-related macular degeneration and lower risk of progression to late disease in large cohort analyses [89,90]. More recent systematic reviews and meta-analyses broadly support this protective association, while also pointing out the persistent limitations of dietary research, including residual confounding and measurement error [91,92].

The biological rationale behind these findings is plausible. Mediterranean-style diets are rich in fiber, polyphenols, and omega-3 fatty acids, all of which may support a more favorable microbiome and higher short-chain fatty acid production [93]. In diabetic retinopathy models, sodium butyrate improved retinal outcomes and was linked to changes in the gut microbiome and related metabolites [94]. In experimental autoimmune uveitis, a diet rich in fermentable fiber altered the intestinal microbiome, reduced permeability, and attenuated ocular inflammation [95]. Population-based data also suggest that diet-related gut microbial indices may be associated with diabetic retinopathy risk, although these findings remain observational [96].

Diet has one clear advantage over more direct microbiome interventions: it is feasible, scalable, and already relevant to overall cardiometabolic health. This makes it especially attractive in diseases such as age-related macular degeneration and diabetic retinopathy, where systemic risk factors already play an important role. At the same time, expectations should remain realistic. Disease-specific ophthalmic effects are likely to be modest, and any benefit will depend heavily on baseline phenotype, adherence, and duration of exposure. Diet may be the most practical entry point into this field, but it is not yet a disease-specific therapeutic tool in any strong clinical sense.

### 5.3. Translational Implications and Future Directions

The future clinical relevance of the gut-eye axis will depend less on broad claims about “the microbiome” and more on better patient stratification and clearer identification of functionally relevant pathways. In glaucoma, for example, lifestyle and dietary research is expanding, but disease-specific evidence remains heterogeneous and still requires stronger validation [97,98]. A recent scoping review likewise concluded that dietary evidence in glaucoma remains heterogeneous and methodologically limited [99].

Another important question concerns the relationship between immune-targeted therapy and the microbiome. In non-infectious uveitis, current pharmacotherapy already overlaps with gut-immune pathways, including Toll-like receptor activity and downstream inflammatory signaling [100]. Experimental work also suggests that antimetabolite drugs can influence intestinal microbiota composition in autoimmune uveitis models [56]. If similar bidirectional interactions are confirmed in humans, they may eventually support a more individualized approach to inflammatory eye disease.

A particularly promising direction is to focus less on whole-community composition and more on microbial function. Metabolite-centered approaches may prove more reproducible than attempts to define one protective or pathogenic taxonomic signature. Short-chain fatty acids, bile acid pathways, and tryptophan-derived metabolites are especially attractive because they are measurable, biologically interpretable, and potentially targetable. They also fit more naturally into clinical trial design than broad compositional endpoints.

Study design remains one of the major challenges. Future cohorts will need repeated sampling over time rather than single cross-sectional measurements. This is especially important because the microbiome is dynamic and may change with diet, treatment, disease activity, and age. Stronger studies will also need to combine microbiome profiling with metabolomics, clinical phenotyping, and carefully documented exposure data [93,94,95,96,97,98,99,101]. Without that level of integration, it will remain difficult to separate stable disease-related signals from background variation. This translational landscape is further complicated by systemic drugs such as metformin, which may overlap with microbiome-related mechanisms and have also been investigated in AMD-focused clinical literature [101].

Clinical implementation will also be difficult unless the field moves toward more standardized endpoints. At present, studies vary widely in their microbiome methods, intervention protocols, and ophthalmic outcome measures. This makes comparison across centers difficult and weakens translational confidence. A microbiome-based approach is therefore unlikely to replace standard ophthalmic treatment in the foreseeable future. Its more realistic short-term role may lie in stratification, adjunctive care, and the identification of subgroups with stronger inflammatory or barrier-related signatures. Over time, this may help refine prevention strategies and support more targeted therapeutic development, but the field is not yet at the stage of routine clinical application.

## 6. Conclusions

Current evidence supports a contributory role of dysbiosis in several ophthalmic diseases, but the strength of that association differs across conditions. The most convincing data are available for age-related macular degeneration and diabetic retinopathy, where clinical observations, mechanistic studies, and, in the case of age-related macular degeneration, genetic analyses all point in a similar direction. In uveitis, the gut-eye axis aligns well with immune-based disease biology and is strongly supported by experimental work. In glaucoma and ocular surface disease, the evidence is growing, especially in relation to neuroinflammation, mucosal immunity, and microbial metabolites, but much of it remains associative. Across the field, the most consistent signals are functional rather than taxonomic. Short-chain fatty acids, barrier dysfunction with endotoxin-related signaling, bile acid pathways, and tryptophan-derived metabolites appear more reproducible than single disease-associated taxa. This makes them attractive targets for future translational research. Even so, major challenges remain. The literature is still limited by cohort heterogeneity, methodological variability, and incomplete control of confounding factors.

Future progress will depend on longitudinal studies, integrated multi-omics approaches, and well-controlled intervention trials. In the near term, the most useful clinical role of the gut-eye axis may be in stratification and adjunctive care. In the longer term, a more precise understanding of microbial function may help bring microbiome-informed approaches into ophthalmic medicine in a meaningful and reproducible way. A central contribution of this review is the integration of multiple ophthalmic diseases within a shared functional framework rather than a disease-by-disease catalog of microbiome associations. This perspective highlights recurring mechanisms, especially metabolite signaling, barrier dysfunction, and immune regulation, and may be more useful for future translational work than isolated taxonomic observations alone.

## Figures and Tables

**Table 1:** Structured summary of current evidence for the gut-eye axis across major ophthalmic diseases. Evidence level was assigned qualitatively based on the breadth and consistency of the available evidence, including human observational studies, genetic analyses, experimental models, and mechanistic coherence, together with the main limitations affecting interpretation.

Disease	Evidence Level	Main Mechanisms	Main Limitations
Age-related macular degeneration	Strong	Chronic inflammation, altered microbial metabolites, immune dysregulation, angiogenic susceptibility	Mostly cross-sectional human data; residual confounding by age, diet, and cardiometabolic status
Diabetic retinopathy	Moderate to strong	Barrier dysfunction, endotoxin-related signaling, reduced SCFA-related protection, bile acid signaling, vascular inflammation	Major treatment-related confounding; many studies remain associative
Uveitis	Moderate	Th17/Treg imbalance, immune priming, altered inflammatory thresholds	Small and heterogeneous human cohorts; substantial treatment confounding
Glaucoma	Emerging	Neuroinflammation, T-cell priming, reduced butyrate-producing taxa, tryptophan metabolite signaling	Human evidence remains mainly associative; possible confounding from chronic topical therapy
Dry eye disease	Moderate	Ocular surface dysbiosis, mucosal immune dysregulation, epithelial stress, SCFA-dependent effects	Low-biomass sampling challenges; contamination risk; small intervention studies
Sjögren’s syndrome	Moderate	Gut dysbiosis, Th17/Treg imbalance, altered metabolite signaling, mucosal immune dysfunction	Confounding by systemic autoimmunity and treatment exposure; limited longitudinal data

## Data Availability

No new data were created or analyzed in this study.

## Abbreviations

The following abbreviations are used in this manuscript: AMD, age-related macular degeneration; AHR, aryl hydrocarbon receptor; BRB, blood-retinal barrier; DED, dry eye disease; DR, diabetic retinopathy; FFAR2/3, free fatty acid receptor 2/3; FOXP3, forkhead box P3; HDAC, histone deacetylase; HLA, human leukocyte antigen; IL, interleukin; IOP, intraocular pressure; LPS, lipopolysaccharide; MR, Mendelian randomization; NLRP3, NOD-like receptor protein 3; RPE, retinal pigment epithelium; SCFA, short-chain fatty acid; STING, stimulator of interferon genes; TGR5, Takeda G protein-coupled receptor 5; Th17, T helper 17 cell; TLR, toll-like receptor.
